# Intragenomic Polymorphism of the ITS 1 Region of 35S rRNA Gene in the Group of Grasses with Two-Chromosome Species: Different Genome Composition in Closely Related *Zingeria* Species

**DOI:** 10.3390/plants9121647

**Published:** 2020-11-25

**Authors:** Alexander V. Rodionov, Alexander A. Gnutikov, Nikolai N. Nosov, Eduard M. Machs, Yulia V. Mikhaylova, Victoria S. Shneyer, Elizaveta O. Punina

**Affiliations:** 1 Laboratory of Biosystematics and Cytology, Komarov Botanical Institute of the Russian Academy of Sciences, 197376 St. Petersburg, Russia; avrodionov@binran.ru (A.V.R.); NNosov@binran.ru (N.N.N.); emachs@binran.ru (E.M.M.); YMikhaylova@binran.ru (Y.V.M.); EPunina@binran.ru (E.O.P.); 2 Biological Faculty, St. Petersburg State University, 199034 St. Petersburg, Russia; 3 Department of Genetic Resources of Oat, Barley, Rye, N.I. Vavilov Institute of Plant Genetic Resources (VIR), 190000 St. Petersburg, Russia; a.gnutikov@vir.nw.ru

**Keywords:** taxonomy, evolutionary genomics, grasses, allopolyploidy, *Zingeria*

## Abstract

*Zingeria* (Poaceae) is a small genus that includes *Z. biebersteiniana*, a diploid species with the lowest chromosome number known in plants (2n = 4) as well as hexaploid *Z. kochii* and tetraploid *Z. pisidica*, and/or *Z. trichopoda* species. The relationship between these species and the other low-chromosomes species *Colpodium versicolor* are unclear. To explore the intragenomic polymorphism and genome composition of these species we examined the sequences of the internal transcribed spacer 1 of the 35S rRNA gene via NGS approach. Our study revealed six groups of ribotypes in *Zingeria* species. Their distribution confirmed the allopolyploid nature of *Z. kochii*, whose probable ancestors were *Colpodium versicolor* and *Z. pisidica*. *Z. pisidica* has 98% of rDNA characteristic only for this species, and about 0.3% of rDNA related to that of *Z. biebersteiniana*. We assume that hexaploid *Z. kochii* is either an old allopolyploid or a homodiploid that has lost most of the rRNA genes obtained from *Z. biebersteiniana*. In *Z. trichopoda* about 81% of rDNA is related to rDNA of *Z. biebersteiniana* and 19% of rDNA is derived from *Poa diaphora* sensu lato. The composition of the ribotypes of the two plants determined by a taxonomy specialist as *Z. pisidica* and *Z. trichopoda* is very different. Two singleton species are proposed on this base with ribotypes as discriminative characters. So, in all four studied *Zingeria* species, even if the morphological difference among the studied species was modest, the genomic constitution was significantly different, which suggests that these are allopolyploids that obtained genomes from different ancestors.

## 1. Introduction

Interspecific hybridization, often accompanied by the formation of allopolyploid genomes, allows plants to bypass the prohibitions on sympatric speciation known from the synthetic theory of evolution [1,2]. Various genetic processes, such as the loss of all or part of the chromosomes of one of the parents, the expansion of transposons, translocations and transpositions, and the loss of a part of the genes of one or both parental genomes, take place in complicated allopolyploid genomes [3,4]. Interspecific hybridization creates unique opportunities for effective positive selection and can be accompanied by saltation speciation [5,6].

An astonishing example of the formation of alloploid genomes has been found in the small genus of annual grasses *Zingeria* P.A. Smirn. One of the species has the lowest chromosome number in angiosperms. The diploid species *Zingeria biebersteiniana* (Claus) P.A. Smirn. has only four chromosomes (2n = 2x = 4) [7]. Two tetraploid species *Z. pisidica* (Boiss.) Tutin and *Z. trichopoda* (Boiss.) P.A. Smirn. have uncertain taxonomic status. Some taxonomists regard them as conspecific, while others believe that there are two separate species. Their ranges of distribution overlap. *Z. trichopoda* occurs in Caucasus, Minor Asia, Syria, Iran, and Iraq; *Z. pisidica* occurs in Caucasus, Minor Asia, and Romania. Several distinguishing morphological characters are described [8], but both species are rather rare, and collected samples are few in number. The sample of tetraploid *Zingeria* collected in Jermuk, Armenia, has been shown to be allopolyploid [9]. One its subgenome is related to *Z. biebersteiniana*, and the other, of unknown origin [9]. Hexaploid *Z. kochii* (Mez) Tzvelev, an endemic species from Armenia is also allopolyploid with karyotype 2n = 12 [10,11]. Its first subgenome comes from *Z. biebersteiniana*, the second subgenome derived from *Colpodium versicolor* (Steven) Schmalh., and the third subgenome is not identified [11].

*Colpodium versicolor* is another grass species that has only four chromosomes in its karyotype [12,13,14,15,16]. Based on morphological characters it is suggested that *C. versicolor* is closely related to *Z. biebersteiniana* [10]. Despite this, *Zingeria* and *Colpodium* were included in the separate tribes Aveneae and Poeae, respectively [17]. Molecular phylogenetic studies have proven that *Zingeria* and *Colpodium* are closely related genera within the subtribe Coleanthinae of tribe Poeae [11,18,19,20,21,22]. It was shown that another closely related to *Zingeria* taxon is *Catabrosella araratica* (Lipsky) Tzvelev [9,11,18,19,20].

The genus *Zingeria* represents a polyploid series including the species with the lowest chromosome number in angiosperms (and rather large chromosomes convenient for cytogenetic studies), and can serve as a good model for speciation and evolution studies, so it is important to understand relationships in this group.

The main approach which allows to determine origin of alloploids is cytogenetic experiments, first of all GISH [9,11]. Cytogenetic studies require viable seeds, which are often not easily accessible, especially the seed of rare and endemic plants like *Zingeria* species. The new sequencing approach (NGS—next-generation sequencing) opens the opportunity to find intragenomic polymorphism resulting from interspecific hybridization, using a small amount of herbarium material. The analysis of rDNA intragenomic polymorphism reveals the hybrid origin of species in *Cypripedium* [23] and *Sparganium* [24]. There are hundreds to thousands tandem copies of rRNA genes in each plant genome, each copy of rDNA operon includes 5′ ETS (external transcribed spacer), 18S rRNA gene, ITS1 (internal transcribed spacer), 5.8S rRNA gene, ITS2, 25S rRNA gene, and 3′ ETS [25]. On rDNA RNA polymerase I transcribes 35S pre-rRNA, which is cleaved by nucleases into 18S, 5.8S, and 25S rRNAs [25]. 35S rDNA loci are an important evolutional marker, and the number of rDNA loci shows significant positive correlation with ploidy level [26]. The most informative regions of 35S rDNA are ITS1 and ITS2 because they have the highest level of intragenomic diversity [27].

The main aim of this work is to investigate genomic origin and subgenomic composition of *Zingeria* species and closely related *Catabrosella araratica* and *Colpodium versicolor* via the targeted NGS of the ITS1 region of 35S rDNA. Herbarium vouchers of *Z. trichopoda* and *Z. pisidica* used for the analyses were identified by agrostologist Tzvelev and completely met the morphological criteria of this species.

## 2. Results

The fragments of 35S rDNA including the 3′-end of 18S rRNA gene, the complete sequence of ITS1 region, and the 5′-end of 5.8S rRNA gene were obtained for six closely related species: four *Zingeria* species, *Colpodium versicolor*, and *Catabrosella araratica*. The total amount of Illumina reads was ca. 20,000 for each species. The length of processed and aligned fragments was 310 bp. We revealed eight ITS1 ribotypes. Six ribotypes were shared between *Zingeria* and *Colpodium* species and two ribotypes were found in studied *Catabrosella* species.

**Figure 1:**
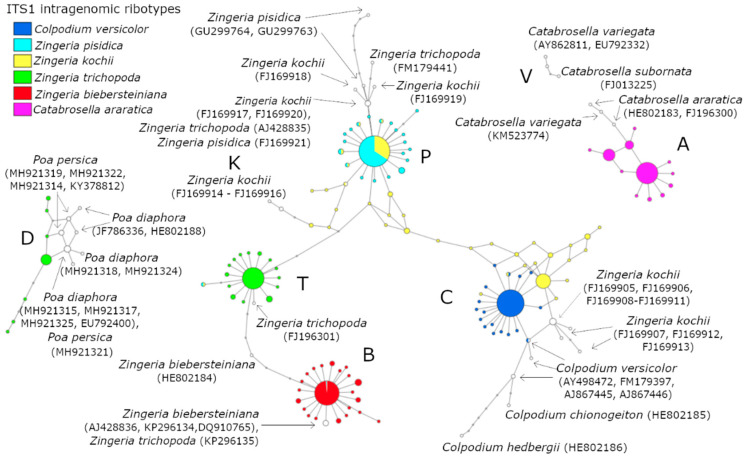
System of ITS1 ribotypes of *Zingeria*, *Colpodium versicolor*, and *Catabrosella*. The filled circles represent intragenomic ribotypes (A, B, C, D, K, P, T, and V), obtained via next-generation sequencing (NGS). The open circles represent sequences from GenBank.

**Figure 2:**
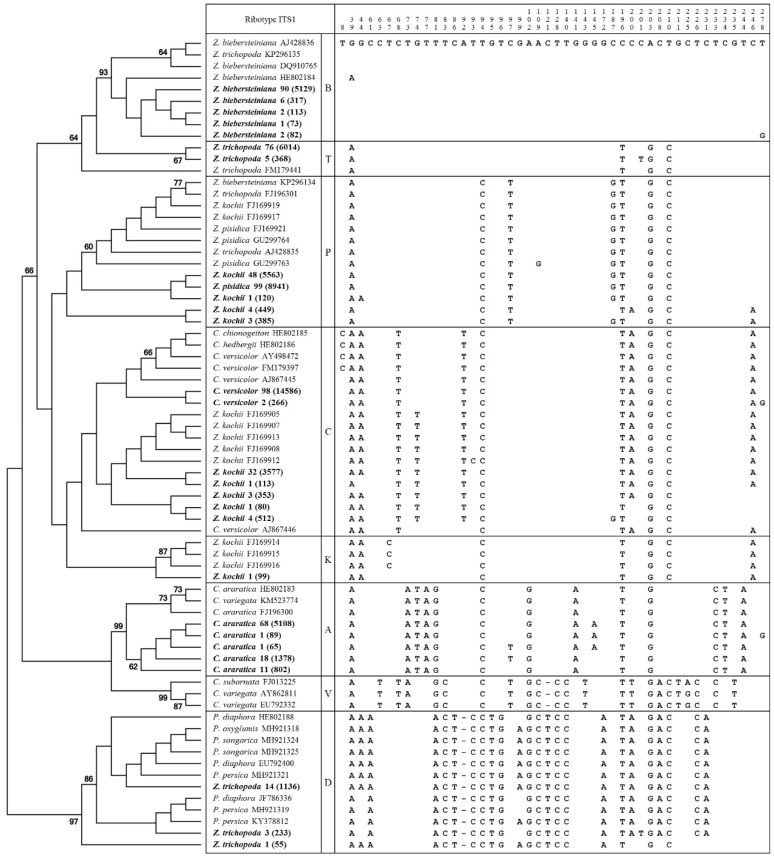
Cladogram and polymorphic nucleotides of ITS1 ribotypes of *Zingeria*, *Colpodium versicolor*, and *Catabrosella*. The percentage of replicate trees in which the associated taxa clustered together in the bootstrap (BS) test are shown next to the branches. The intragenomic ribotypes obtained via NGS are in bold, followed by percentage of the ribotype in the genome and total number of corresponding reads in the brackets.

Ribotype A (Figure 1 and Figure 2) included intragenomic variants of ITS1 from *Catabrosella araratica* (Lipsky) Tzvelev. This ribotype included all sequences of *Catabrosella araratica* from GenBank and formed a separated network, not connected with ribotypes of *Zingeria* and *Colpodium* (Figure 1). The sequence of *Catabrosella variegata* subsp. *variegata* KM523774 (Adygea, Russia, voucher “US:Soreng et al. 8040”) [28] was also located in this network. The observed position of KM523774 could be explained by the misidentification of voucher specimen, and the specimen should be identified as *C. araratica*. Other ITS sequences of *C. variegata* as well as *C. subornata* formed another network and belong to ribotype V (Figure 1).

Ribotypes B, C, T, K, and P joined into one network (Figure 1). Ribotype B was characteristic for *Z. biebersteiniana*. Minor quantity (0.2%) of ribotype B was found in the *Z. pisidica* genome (Figure 1). Ribotype C was found in *Colpodium versicolor*, whose ITS1 intragenomic variants consisted of 98% of ribotype C. This ribotype was closely related to sequences of *Colpodium hedbergii* and *Colpodium chionogeiton*. Besides *Colpodium versicolor* ribotype C was presented in the *Z. kochii* genome. The genome of *Z. kochii* had about 40% of ITS1 variants belonging to the ribotype C (Figure 1 and Figure 2). Ribotype K consisted of ITS sequences from cloned sequenced of *Z. kochii* (FJ1699914, FJ169915 and FJ169916, [11]) and one rare variant of ITS1, obtained from *Z. kochii* via NGS. Ribotype P included about 99% of intragenomic ITS1 variants of *Z. pisidica* (Figure 1). Also, this ribotype was found in *Z. kochii* and *Z. trichopoda* (two sequences from GenBank).

Two different ribotypes, T and D, were found in the allopolyploid *Z. trichopoda* genome. There were about 81% of the ribotype T and 18% of the ribotype D in *Z. trichopoda*. The ribotype T is a closely related ribotype to a derivate of ribotype B obtained from *Z. biebersteiniana* HE802184 [29]. Surprisingly, our NGS analysis revealed that the second ribotype of *Z. trichopoda* was distant from both the ribotype B of *Z. biebersteiniana* and the ribotype C of *Colpodium* s. str. The sequences of this ribotype D were related to *Poa diaphora* (Figure 1 and Figure 2). We found the D ribotype only in *Z. trichopoda* and did not see any sign of it in the genomes of other *Zingeria* species, *Colpodium versicolor* or *Catabrosella araratica*.

**Figure 3:**
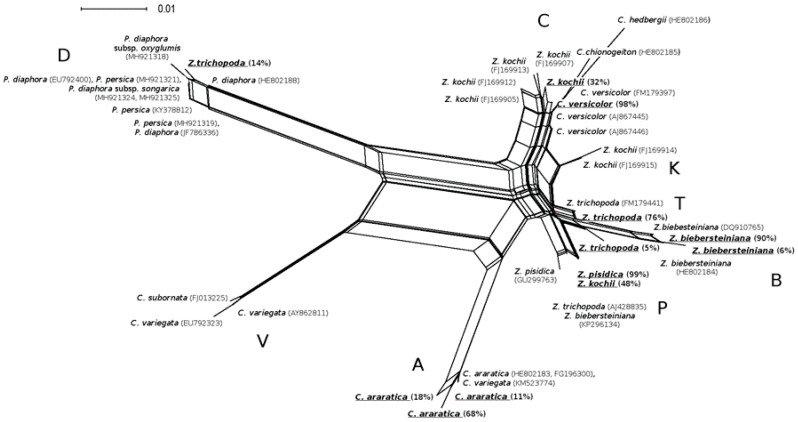
Network among ITS1 ribotypes of grasses from the *Zingeria*, *Colpodium*, and *Catabrosella* genera, revealed by the split decomposition algorithm. Intragenomic ITS ribotypes obtained via NGS are underlined and followed by percentage of the ribotype in genome. Sequences from GenBank followed by accession number. Groups of ribotypes are labeled by capital letters A, B, C, D, K, P, T, and V.

All main ribotypes could be identified on the phylogenetic tree (Figure 2). All sequences of *Zingeria* fell within the same clade except for the second subgenome of *Z. trichopoda* that was related to *P. diaphora* group (=genus *Eremopoa*). All ITS1 reads of *Z. biebersteiniana* and 81% ITS1 reads of *Z. trichopoda* formed a single clade with ITS-sequences of *Z. biebersteiniana* from GenBank (BS=64). *Z. trichopoda* had 17–18% of ITS1 sequences closely related (BS=97 and 86) to ITS1 sequences of the *P. diaphora* aggregate species. The phylogenetic tree of the ITS1 sequences (Figure 2) was in congruence with the network data (Figure 3).

The phylogenetic tree (Figure 2) was based on the evolution models, implying a gradual accumulation of mutations followed by dichotomous branching of phylogenetic trees. However, the evolutionary scenarios of the allopolyploid plants imply events of backcrossing. Therefore, we used the Neighbor-net algorithm implemented in the program SplitsTree4, suggested for the reconstruction of reticulate evolution [30]. The Neighbor-net algorithm builds a network called a split graph. The split graph (Figure 3) shows several possible ways of grouping DNA sequences with varying degrees of probability, known as “splits”, and reflects the presence of *homoplasy* in the data. The reticulate network provides a picture of evolution of the rRNA genes of the two-chromosome grasses. In the network, the edges represent lineages of descent or reticulate events such as hybridization, conversion or crossing-over. All nodes of the network correspond to hypothetical ancestors, whether the product of speciation and mutation, or hybridization or recombination events [30]. On the network there were eight ribotypes among the studied species. Ribotypes P and B were closely related, and ribotype K seems to be a result of recombination between ribotypes B and C (Figure 3).

## 3. Discussion

NGS allow us to reveal intragenomic polymorphism in *Zingeria* polyploid species and clarify the relationships between *Zingeria*, *Colpodium versicolor* and *Catabrosella araratica*. A species *C. araratica*, having ribotype A, was originally described as *C. araratica* Lipsky, type in LE (BIN RAS SPb.) “On the northern slope of Mount Bolshoi Ararat, 8. VII 1893b V. Lipsky”. Isotype at LE. Later, Woronov proposed to attribute this species to the genus *Colpodium* sensu lato [31], then Tzvelev [32], reforming *Colpodium*, assigned this species to a special section in the genus *Catabrosella*, *C. araratica*, sect. Nevskia. Later, we showed that this species is only distantly related to other species of the genus *Catabrosella* and differs from it in morphology, and therefore deserves to be separated into a new genus *Nevskia* [19,20]. Recently, Tkach et al. came to a similar conclusion, proposing a new generic and specific name *Hyalopodium araraticum* (Lipsky) Röser & Tkach [33]. If the determination of the number of chromosomes in this species 2n = 42 [34] is correct, it seems possible that *Catabrosella araratica* is an “old” hexaploid, in which rDNA exhibit low level of intragenomic polymorphism (Figure 1), and its rDNA are isogenized.

The reticulate evolution could be the reason for the very complicated relationship between species in the genus *Zingeria* revealed by the obtained results. Several independent acts of interspecific hybridization led to the emergence of *Z. kochii* and plants that were determined by Tzvelev as *Z. pisidica* and *Z. trichopoda*. The hexaploid *Z. kochii* appears to have arisen relatively recently as the result of hybridization between *C. versicolor* and *Z. pisidica*. Our observations of ITS1 intragenomic polymorphism are in good agreement with the GISH results [11]. The origin of *Z. pisidica* remains unclear—apparently, it is a diploid or an old allopolyploid, 99% of modern rDNA belongs to an unknown ancestor from the *Zingeria biebersteiniana* circle of kinship (Figure 1 and Figure 3).

*Zingeria trichopoda* has a completely different origin. What the differences are between *Z. pisidica* and *Z. trichopoda* is not entirely clear. The species *Agrostis pisidica* (= *Z. pisidica*) was described by Boissier based on plants collected by Colonel Tchihatcheff in Turkey (Anatolia) [35]. Samples of *Milium trichopodum* (= *Z. trichopoda*) were collected in Syria [17,36]. The chromosomal numbers of *Zingeria* specimens from Asia Minor are unknown.

First, tetraploid *Zingeria* was recorded in Romania [37]. Hackel was the first who has shown the relationship of the species from Romania with one found in Russia, *Agrostis biebersteiniana* Claus (= *Z. biebersteiniana*) and named it *A. biebersteiniana* Claus var. *densior* Hack., however, Grecescu believed that it was an endemic species of the Romanian Plain, *A. densior* (Hack.) Grecescu [38]. Then Schischkin [39] classified the Romanian samples as *A. pisidica* Boiss. (= *Z. pisidica*), a species described by Boissier [36] from Anatolia (Turkey). Later, Chrtek [40] included *A. densior* in the genus *Zingeria* as *Z. densior* (Hack.) Chrtek. Tutin [41] and modern Romanian botanists [42] believe that this species should be called *Z. pisidica* (Boiss.) Tutin. Tzvelev, initially assuming that *Z. pisidica* and *Z. trichopoda* are synonyms, considered the Romanian *Zingeria* to be *Z. trichopoda* (Boiss.) P.A. Smirn. [17]. According to Tzvelev [43] and Gabrielian [8], these species differ in the structure of panicles—in *Z. trichopoda*, they are on average larger, more spreading, with thinner branches and longer spikelets (5–13 mm) in comparison with shorter ones (0.7–5 mm) in *Z. pisidica*.

It was shown that *Zingeria* samples collected in Georgia and in the Sisian and Jermuk regions of Armenia, morphologically identified as *Z. trichopoda* and/or *Z. pisidica*, have 2n = 8 [9,10,11,12,13,20,44,45]. *Z. pisidica* and/or *Z. trichopoda*, both of these species or one of them, have been reported as tetraploid in “Grasses of U.S.S.R.” [17], “Flora Europaea” [41], “Flora of the Caucasus” [43,46], and “Grasses of Russia” [47]. However, few diploid *Zingeria* sp. samples, 2n = 4, morphologically indistinguishable from the tetraploid race of *Z. trichopoda*/*Z. pisidica* were collected in the Sisian region of Armenia and in Nakhchivan Autonomous Republic of Azerbaijan [12,44].

It was shown by GISH that one of subgenomes of the tetraploid *Zingeria* collected in Jermuk, Armenia, is related to *Z. biebersteiniana*, and the other is of unknown origin designated as Z.trichopoda [9]. Later the authors note [11] that the material specified as *Z. trichopoda* in cytogenetic studies [7,9,14] according more recent classifications [43,46] belongs to *Z. pisidica*. Formely *Z. pisidica* had been regarded as part of *Z. trichopoda* [17], but in later taxonomical treatments Tzvelev [43,46] accepts both *Z. trichopoda* and *Z. pisidica* at species rank. 

In the genome of *Z. trichopoda* we see two ribotypes of completely different origins: there are about 81% of the ribotype T and 18% of the ribotype D. The ribotype T was inherited from a species from the kinship circle of two-chromosomal grasses, but the ribotype D could only originate from a species of *Eremopoa* (= the *Poa diaphora* aggregate). How this could have happened remains unclear, since *Poa diaphora* has the basic number of chromosomes x = 7. *Poa diaphora* Trin., known for a long time as *Eremopoa altaica* (Trin.) Roshev., was a type species of the genus *Eremopoa* Roshev. [48]. It was shown that the divergence of species in the genus is associated with polyploidy: *Eremopoa oxiglumis* (Boiss.) Roshev. and *E. persica* (Trin.) Roshev. are diploids (2n = 14) [44,49,50,51,52,53,54]. *Eremopoa songarica* (Schrenk) Roshev. is a species of tetraploid origin (2n = 28) [44,50,51,52,53,54], and *E. altaica* (Trin.) Roshev. is hexaploid (2n = 42) [44,50,53,54]. After *Eremopoa* was described, most authors accepted the genus [17,41,47,55,56], however, a comparison of chloroplast *trn*T-*trn*L-*trn*F and nuclear ITS-sequences showed that *Eremopoa* lies among *Poa* species on the phylogenetic tree [21,22,57,58,59,60,61]. To date, *P. diaphora* has been joined with the species of genus *Lindbergia* Lehm. ex Link & Otto and *P. speluncarum* J.R.Edm. as *Poa* of subgenus *Pseudopoa* [58].

Until now we have known only of one case where species with radically different karyotypes have given viable offspring; these are the hybrids of two deer species, the Indian muntjac *Muntiacus munjak vaginalis* Zimmermann 1780 (2n = 6♂ and 7♂) and the Reeves’ muntjac M. reevesii Ogilby 1839 (2n = 46). These hybrids have 2n = 26 for the female and 2n = 27 for the male and are likely to be sterile [62,63,64]. It can be assumed that hybrids between *Poa diaphora* aggr. (x = 7) and another hypothetical ancestor of *Z. trichopoda* having x = 2 could be reproduced in the first generations only vegetatively, at this time there was a crossing over or conversion, or translocation with the transfer of ribotype D rDNA to the chromosomes of the bichromosomal genome. Then, gradually or saltationally, there was a loss of all *Poa* chromosomes and a duplication of low-chromosome-number-ancestor chromosomes, as often happens in distant hybrids [3,4,65].

In 1984, Löwe proposed a genomic criterion for separating species [66]. Here, we show that even morphologically slightly different plants from the same genus can have different sets of rDNAs, which suggests that these are allopolyploids with different genomic constitutions and different ancestors.

Differences in the organization of genomes between the samples determined in the Herbarium LE as *Z. trichopoda* and *Z. pisidica* are very significant. Along with this the relationship between the samples from the Transcaucasian flora, described initially as *Z. trichopoda* [12,17,44], and later as two species *Zingeria trichopoda* and *Z. pisidica* [43,46,47] are very confusing and vague. For these reasons, we would suggest to describe the plants studied by us as two new singleton species based on a new uniquely determined criterion: a fundamental difference in the composition of their ribotypes.

*Zingeria tzvelevii* Rodionov, Gnutikov, Nosov-sp. nov Type: *Z. pisidica*: Georgia: Samtskhe–Javakheti region (mkhare), Ninotsminda District, the coast of Hanchali lake. 12-Jul-1960. Coll. S. K. Cherepanov, N. N. Tzvelev. Det. N. N. Tzvelev; diploid or tetraploid species, ribotype P, ancestor of *Z. kochii*.

*Zingeria probatovae* Rodionov, Gnutikov, Nosov-sp. nov Type: *Z. trichopoda*: Georgia: Samtskhe–Javakheti region (mkhare), Borjomi District, near of the Tabithuri Lake. 27-Jun-1980. Coll. T. Popova, Y. Menitzkiy. Det. N. N. Tzvelev; 2n = 8, ribotypes T and D, whose rDNA contains *Zingeria* rDNA and *Poa diaphora* rDNA.

Singleton species are very common in biodiversity samples, and their discovery is very important [67]. We hope this will further help to untangle the complexities of the case of the *Zingeria* spp. of Caucasus.

## 4. Materials and Methods

The following plant materials were used in this study: *Z. biebersteiniana* (Rakhinka settlement, Sredneakhtubinsky District, Volgograd Oblast, Russia, N 49.00972°, E 44.91166°, collected on 21 June 2016 by E. Punina and A. Rodionov); *Z. kochii* (the left riverside of the Akhuryan River, Gyumri, Shirak, Armenia, collected on 7 July 1960 by S. K. Cherepanov and N. N. Tzvelev, identified by N. N. Tzvelev); *Z. pisidica* (the coast of lake Hanchali, Ninotsminda District, Samtskhe–Javakheti, Georgia, collected on 12 July 1960 by S. K. Cherepanov and N. N. Tzvelev, identified by N. N. Tzvelev); *Z. trichopoda* (lake Tabithuri, Borjomi, Samtskhe–Javakheti, Georgia, collected 27 July 1980 by T. Popova and Y. Menitzkiy, identified by N. N. Tzvelev); *Catabrosella araratica* (Aragats Mount, Aragatsotn, Armenia, 3300 m alt., collected by E. Gabrielyan, identified by A. Ghukasyan); *Colpodium versicolor* (the source of the Nazylykol River, Teberda Nature Reserve, Karachay-Cherkessia, Russia, 2500 m alt., collected on 21 August 2003 by E. Punina, A. Rodionov, S. Bondarenko, and Y. Punin, identified by N. N. Tzvelev). All voucher specimens were deposited in the Herbarium of the Komarov Botanical Institute in St. Petersburg, Russia (LE).

The total genomic DNA was extracted from the herbarium material by the CTAB method [68] with small modifications or using Qiagen DNeasy Plant Mini Kit (Qiagen Inc., Valencia, CA, USA). The DNA extraction was done in The Core Facilities Center “Cell and Molecular Technologies in Plant Science” at the Komarov Botanical Institute RAS (St. Petersburg, Russia).

Library preparation and Illumina MiSeq sequencing were performed at Center of Collective Usage of All-Russia Institute for Agricultural Microbiology. Marker sequences (3′ part of 28S rRNA gene, complete sequences of ITS1 and 5′ part of 5.8S rRNA gene) were amplified using primers ITS1P [69] and ITS2 [70]. The raw sequencing data were processed using software tools FastQC [71], Trimmomatic [72] and Fastq-join [73]. Plant ribotypes were sorted by frequency and filtered from fungi and bacteria using BLAST [74]. Selected ribotypes we aligned by MUSCLE algorithm [75] in MEGA 7.0 [76]. In addition to ribotype data we included in the analysis sequences from GenBank for *Zingeria*, *Colpodium*, *Catabrosella*, *Poa diaphora*, and *Poa persica* (Table S1).

We estimated the relationships between ribotypes using the statistical parsimony method [77] implemented in the software TCS 1.21 [78]. This method allows us to collapse the sequences into haplotypes, calculates an absolute distance matrix for all pairwise comparisons of haplotypes and parsimony connection limit, and constructs haplotype networks. For the analysis, we chose the e 95% cut-off for the probabilities of parsimony for mutational steps. The obtained network was visualized in TCSBU [79,80] (https://cibio.up.pt/software/tcsBU/). Neighbor-net splits decomposition method implemented in SplitsTree 4 [30] was used to check the relative interaction between ribotypes. This method is proposed for the study of network evolution of genomes [30,81]. The maximum likelihood (ML) phylogenetic analysis was performed with MEGA 7.0 using the Kimura-2 substitution model, which was chosen using BIC method. Bootstrap analysis was performed with 1000 replicates.

## Supplementary Materials

The following are available online at https://www.mdpi.com/2223-7747/9/12/1647/s1, Table S1: Taxon names, GenBank accession numbers and sources of the ITS sequences used in this study.
