# Correction: Rodionov, A.V., et al. Intragenomic Polymorphism of the ITS 1 Region of 35S rRNA Gene in the Group of Grasses with Two-Chromosome Species: Different Genome Composition in Closely Related *Zingeria* Species. *Plants* 2020, *9*, 1647

**DOI:** 10.3390/plants10030463

**Published:** 2021-03-01

**Authors:** Alexander V. Rodionov, Alexander A. Gnutikov, Nikolai N. Nosov, Eduard M. Machs, Yulia V. Mikhaylova, Victoria S. Shneyer, Elizaveta O. Punina

**Affiliations:** 1 Laboratory of Biosystematics and Cytology, Komarov Botanical Institute of the Russian Academy of Sciences, 197376 St. Petersburg, Russia; avrodionov@binran.ru (A.V.R.); NNosov@binran.ru (N.N.N.); emachs@binran.ru (E.M.M.); YMikhaylova@binran.ru (Y.V.M.); EPunina@binran.ru (E.O.P.); 2 Biological Faculty, St. Petersburg State University, 199034 St. Petersburg, Russia; 3 Department of Genetic Resources of Oat, Barley, Rye, N.I. Vavilov Institute of Plant Genetic Resources (VIR), 190000 St. Petersburg, Russia; a.gnutikov@vir.nw.ru

The authors wish to make the following corrections to their paper [1].

In Section 2. (Results), Paragraph 2, Page 3 of 11: “The sequence of *Catabrosella variegata* subsp. *variegata* KM523774 (Adygea, Russia, voucher “US:Soreng et al. 8040”) [28] was also located in this network. The observed position of KM523774 could be explained by the misidentification of voucher specimen, and the specimen should be identified as C. *araratica*.” should be deleted.

In Figure 1, *Catabrosella variegata* KM523774 should be replaced by *Catabrosella araratica* HE802183.

In Figure 2, *Catabrosella variegata* KM523774 should be replaced by *Catabrosella araratica* HE802183.

In Figure 3, *Catabrosella variegata* KM523774 should be deleted.

In Supplementary Table S1, line “C. variegata KM523774 Adygea, Russia [8]” and reference 8: “8 Soreng, R.J.; Gillespie, L.J.; Koba, H.; Boudko, E.; Bull, R.D. Molecular and morphological evidence for a new grass genus, Dupontiopsis (Poaceae tribe Poeae subtribe Poinae s.l.), endemic to alpine Japan, and implications for the reticulate origin of Dupontia and Arctophila within Poinae s.l. J. Syst. Evol. 2015, 53, 138–162, doi:10.1111/jse.12146.” should be deleted.

In Supplementary Table S1, references 9 and 10 should be considered as references 8 and 9, respectively.

The changes do not affect the main results and conclusion of the paper. We would like to apologize for any inconvenience caused to readers. We would like to apologise to Prof. Robert Soreng, whom we improperly mentioned in the text. The manuscript will be updated and the original will remain available on the article webpage.
