# Supplementary material for: Intragenomic Polymorphism of the ITS 1 Region of 35S rRNA Gene in the Group of Grasses with Two-Chromosome Species: Different Genome Composition in Closely Related Zingeria Species
Source: Plants (Basel). 2020 Nov 25;9(12):1647. doi: 10.3390/plants9121647 (PMC7760792; doi:10.3390/plants9121647)

Supplement. Table S. Taxon names, GenBank accession numbers and sources of the ITS sequences used in this study

Species	Number in GenBank	Specimen	Reference
Zingeria biebersteiniana (Claus) P.A. Smirn.	AJ428836	Prishib, Volgograd, Russia	[1]
Z. biebersteiniana	DQ910765	Rakhinka, Volgograd, Russia	[2]
Z. biebersteiniana	HE802184		[3]
Z. biebersteiniana	KP296134	specimen_voucher "HAL:M.Roeser s.n."	Acedo,C., Alonso,A. and Llamas,F. Unpublished
Z. kochii (Mez) Tzvelev	FJ169905	Kotayskiy region, Armenia	[4]
Z. kochii	FJ169906	Kotayskiy region, Armenia	[4]
Z. kochii	FJ169907	Kotayskiy region, Armenia clone 5m	[4]
Z. kochii	FJ169908	Kotayskiy region, Armenia clone 14f	[4]
Z. kochii	FJ169909	Kotayskiy region, Armenia clone 24f	[4]
Z. kochii	FJ169910	Kotayskiy region, Armenia clone 3f	[4]
Z. kochii	FJ169911	Kotayskiy region, Armenia clone 10f	[4]
Z. kochii	FJ169912	Kotayskiy region, Armenia clone 8r	[4]
Z. kochii	FJ169913	Kotayskiy region, Armenia clone 5f	[4]
Z. kochii	FJ169914	Kotayskiy region, Armenia clone 16r	[4]
Z. kochii	FJ169915	Kotayskiy region, Armenia clone 1f	[4]
Z. kochii	FJ169916	Kotayskiy region, Armenia	[4]

		clone 6r	
Z. kochii	FJ169917	Kotayskiy region, Armenia clone 4r	[4]
Z. kochii	FJ169918	Kotayskiy region, Armenia clone 1m	[4]
Z. kochii	FJ169919	Kotayskiy region, Armenia clone 2m	[4]
Z. kochii	FJ169920	Kotayskiy region, Armenia 3m	[4]
Z. pisidica (Boiss.) Tutin	GU299764	Jermuk, Armenia	[4]
Z. pisidica	FJ169921	Jermuk, Armenia	[4]
Z. pisidica	GU299763	Jermuk, Armenia	[4]
Z. trichopoda (Boiss.) P.A. Smirn.	AJ428835	Jermuk, Armenia	[1]
Z. trichopoda	FM179441	(Jermuk, Armenia) Seed obtained from Institute of Plant Genetics and Crop Plant Research, Gatersleben in 2002	[5]
Z. trichopoda	FJ196301	Gori District, Georgia	[2]
Z. trichopoda	KP296135	specimen voucher "HAL:M.Roeser s.n."	Acedo,C., Alonso,A. and Llamas,F. Unpublished
Catabrosella araratica (Lipsky) Tzvelev	HE802183		[3]
C. araratica	FJ196300	Gegharkunik region, Armenia	[2]
Catabrosella subornata E.B. Alexeev	FJ013225	Talysh-Mugan Autonomous Region, Azerbajan	[2]
Catabrosella variegata (Boiss.) Tzvelev	AY862811	Karachaevo-Cherkessiya, Russia	[6]
C. variegata	EU792332	Turkey	[7]
C. variegata	KM523774	Adygea, Russia	[8]
Colpodium chionogeiton (Pilg.) Tzvelev	HE802185		[3]
Colpodium hedbergii (Melderis) Tzvelev	HE802186		[3]
Colpodium versicolor Woronow ex Grossh.	AY497472	Karachaevo-Cherkessiya, Russia	[2]
C. versicolor	FM179397	South Osetia, Georgia	[5]
C. versicolor	AJ867446	Armenia	[4]
C. versicolor	AJ867445	Georgia	[4]
Poa diaphora Trin.	EU792400	Turkey	[7]
P. diaphora	HE802188		[3]

P. diaphora	JF786336	Altai republic, Russia	Nosov,N., Machs,E.M., Rodionov,A.V. Unpublished
Poa diaphora subsp. *oxyglumis* (Boiss.) Soreng & G.H.Zhu	MH921317	Turkey	[9]
Poa diaphora subsp. *oxyglumis*	MH921318	Turkey	[9]
Poa diaphora subsp. *oxyglumis*	MH921315	Turkey	[9]
P. diaphora var. *songarica* (Schrenk ex Fisch. & C.A.Mey.) Soreng, Cabi & L.J.Gillespie	MH921324	Iran	[9]
P. diaphora subsp. *songarica*	MH921325	Turkey	[9]
P. persica Trin.	KY378812	Turkey	[10]
P. persica	MH921319	Iran	[9]
P. persica	MH921321	Iran	[9]
P. persica	MH921322	Iran	[9]
Poa persica subsp. *multiradiata* (Trautv.) Soreng, Cabi & L.J.Gillespie	MH921314	Turkey	[9]

1. Kotseruba, V.; Gernand, D.; Meister, A.; Houben, A. Uniparental loss of ribosomal DNA in the allotetraploid grass Zingeria trichopoda (2 n= 8). *Genome* **2003**, *46*, 156–163, doi:10.1139/g02-104.

2. Kim, E.S.; Bolsheva, N.L.; Samatadze, T.E.; Nosov, N.N.; Nosova, I.V.; Zelenin, A.V.; Punina, E.O.; Muravenko, O. V.; Rodionov, A. V. The unique genome of two-chromosome grasses Zingeria and Colpodium, its origin, and evolution. *Russ. J. Genet.* **2009**, *45*, 1329, doi:10.1134/S1022795409110076.

3. Hoffmann, M.H.; Schneider, J.; Hase, P.; Roser, M. Rapid and recent world-wide diversification of bluegrasses (Poa, Poaceae) and related genera. *PLoS One* **2013**, *8*, E60061, doi:10.1371/journal.pone.0060061.

4. Kotseruba, V.; Pistrick, K.; Blattner, F.R.; Kumke, K.; Weiss, O.; Rutten, T.; Fuchs, J.; Endo, T.; Nasuda, S.; Ghukasyan, A.; et al. The evolution of the hexaploid grass Zingeria kochii (Mez) Tzvel.(2n= 12) was accompanied by complex hybridization and uniparental loss of ribosomal DNA. *Mol. Phylogenet. Evol.* **2010**, *56*, 146–155, doi:10.1016/j.ympev.2010.01.003.

5. Schneider, J.; Döring, E.; Hilu, K.W.; Röser, M. Phylogenetic structure of the grass subfamily Pooideae based on comparison of plastid *matK* gene-3′ *trnK* exon and nuclear ITS sequences. *Taxon* **2009**, *58*, 405–424, doi:10.1002/tax.582008.

6. Rodionov, A. V.; Kim, E.S.; Nosov, N.N.; Raiko, M.P.; Machs, E.M.; Punina, E.O. Molecular phylogenetic study of the genus Colpodium sensu lato (Poaceae: Poeae). *Ecol. Genet.* **2008**, *6*, 34–46, doi:10.17816/ECOGEN6434-46.

7. Gillespie, L.J.; Soreng, R.J.; Bull, R.D.; Jacobs, S.W.L.; Refulio-Rodriguez, N.F. Phylogenetic relationships in subtribe Poinae (Poaceae, Poeae) based on nuclear ITS and plastid trnT-trnL-trnf sequences. *Botany* **2008**, *86*, 938–967, doi:DOI: 10.1139/B08-076.

8. Soreng, R.J.; Gillespie, L.J.; Koba, H.; Boudko, E.; Bull, R.D. Molecular and morphological evidence for a new grass genus, Dupontiopsis (Poaceae tribe Poeae subtribe Poinae s.l.), endemic to alpine Japan, and

implications for the reticulate origin of Dupontia and Arctophila within Poinae s.l. *J. Syst. Evol.* **2015**, *53*, 138–162, doi:10.1111/jse.12146.

9. Gillespie, L.J.; Soreng, R.J.; Cabi, E.; Amiri, N. Phylogeny and taxonomic synopsis of Poa subgenus Pseudopoa (including Eremopoa and Lindbergella) (Poaceae, Poeae, Poinae). *PhytoKeys* **2018**, *111*, 69, doi:10.3897/phytokeys.111.28081.

10. Cabi, E.; Soreng, R.J.; Gillespie, L.J. Taxonomy of Poa jubata and a new section of the genus (Poaceae). *Turk. J. Bot.* **2017**, *41*, 405–415, doi:10.3906/bot-1611-28.

Fig. S1. Network among ITS1 ribotypes of grasses from the *Zingeria, Colpodium and Catabrosell*a genera, revealed by the split decomposition algorithm. Intragenomic ribotypes obtained via NGS are written in capital letters, the number before represents percentage of the ribotype in genome. Sequences from GenBank followed by accession number.

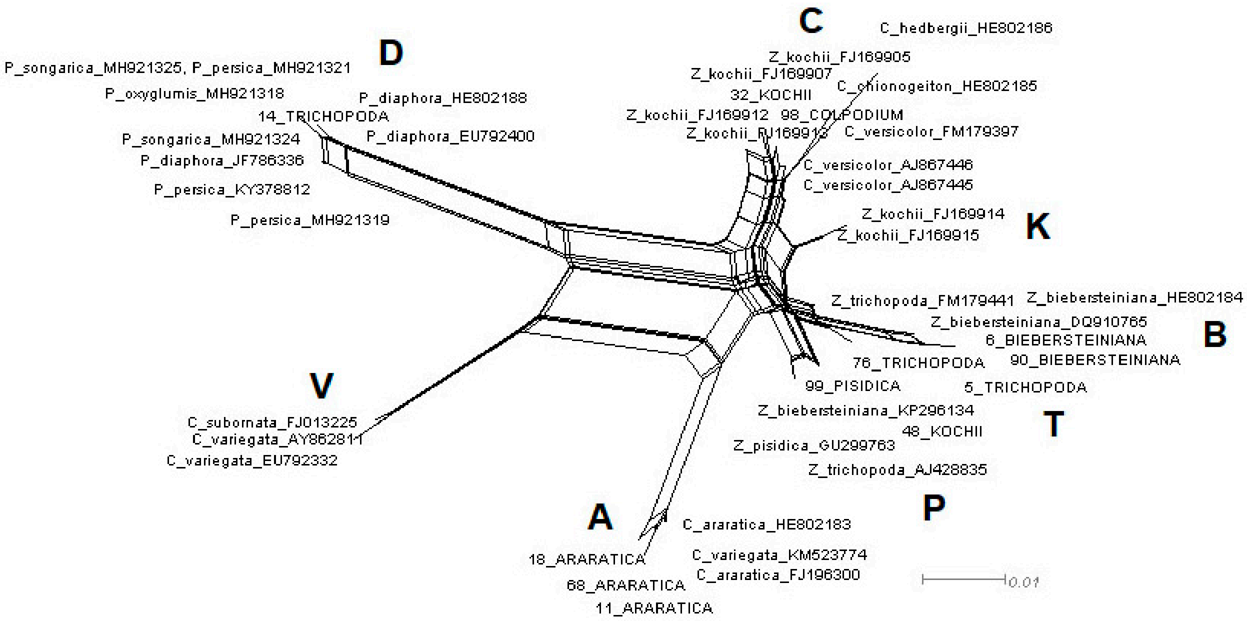

Supplement: Supplementary file 1 [file plants-09-01647-s001.pdf]
